# A Dire Presentation of Carcinoma Cervix in a Human Immunodeficiency Virus-Positive Case: A Salient Display

**DOI:** 10.7759/cureus.45605

**Published:** 2023-09-20

**Authors:** Soumya Pamnani, Sanket S Bakshi, Swarupa Chakole

**Affiliations:** 1 Department of Medicine, Jawaharlal Nehru Medical College, Datta Meghe Institute of Higher Education and Research, Wardha, IND; 2 Department of Community Medicine, Jawaharlal Nehru Medical College, Datta Meghe Institute of Higher Education and Research, Wardha, IND

**Keywords:** human immunodeficiency virus, pap-smear, triple drug regimen, chemo radiotherapy (chemo-rt), carcinoma cervix

## Abstract

Despite being one of the commonest malignancies among women worldwide, carcinoma of the cervix, due to its nonspecific symptoms, goes undiagnosed until it reaches advanced stages. This is especially true among women living with human immunodeficiency virus (HIV) as the rate of screening for them is much less as compared to noninfected women. HIV infection greatly impacts the treatment and the prognosis of the diagnosed carcinoma. The existing common linkage between the occurrence of HIV and that of cervical cancer has some significant common elements such as low socio-economic conditions and poor hygiene. The treatment methods in such cases, prove to be of concern, taking into consideration the seropositive status of the case. Here, we discuss one such case of a seropositive patient who presented with complaints of leukorrhea, dysmenorrhea, and dyspareunia. She had stable vitals, with a pulse rate of 86/minute and blood pressure of 100/80 mmHg. On clinical examination, she was diagnosed with stage International Federation of Gynecology and Obstetrics (FIGO) IIIB cervical carcinoma. Under all aseptic precautions, a cervical biopsy was taken and moderately differentiated squamous cell carcinoma of the cervix was diagnosed. A multidisciplinary approach was decided as the course of action, after which she was referred to the department of medical oncology for chemoradiation. Five cycles with a dose of ten Gray (GY) per cycle were planned with concurrent chemotherapy with cisplatin per week. The patient was advised to follow up in the gynecology outpatient department after completion of her chemoradiation cycles for further evaluation and management.

## Introduction

Cervical cancer is the most common malignancy after breast carcinoma among Indian women [1]. The relationship between cervical cancer and the human immunodeficiency virus (HIV) is complicated [2]. In a low-resource country like India, both HIV and cervical carcinoma are highly prevalent and much more likely to go overlooked [3]. The absence of effective screening and treatment leads to significantly higher morbidity and mortality among such patients [4]. Advanced-stage carcinoma among such patients becomes extremely difficult to treat and follow up, as seen in our case [5,6]. Hence, the prevention of such cases by vigorous and periodic screening is extremely important. Although the effects of HIV on the risk of cervical cancer are widely documented, little is known about how HIV affects cervical cancer survivorship. Results of people with and without HIV infection appear to be comparable in the setting of antiretroviral therapy (ART) in retrospective investigations of anal cancer, which is associated similarly to HPV. Observations, however, have given rise to the possibility that women with HIV may experience worse cervical cancer outcomes. Although trends did not reach statistical significance and designs had little control over potential biases, two registry-based studies have revealed a potential link between HIV and potentially lower cervical cancer survival. HIV infection more than quadrupled the risk of mortality among women receiving curative, guideline-concordant medication, even in the setting of high access to and usage of ART. Early oncologic development in HIV-positive women appeared to be responsible for the extra mortality, with competing mortality from HIV-associated illnesses making up the smallest portion of the excess risk of death. Regardless of HIV status, difficulties were encountered in maintaining and finishing guideline-concordant medication, which most likely contributed to the overall poor survival rate. To combat the increased incidence of cervical cancer in sub-Saharan Africa and other low-income settings, new retention techniques for all women as well as improved treatment methods for those with cervical cancer linked to HIV are urgently needed.

## Case presentation

A 42-year-old woman, with a previous history of three successful pregnancies, presented with complaints of white vaginal discharge, post-coital bleeding, and dyspareunia for the last two months. She also had pain in her left flank and hesitancy during urination. She was diagnosed as infected with HIV, four years before she visited our hospital. She did not give any history of multiple sexual partners, intravenous drug use, or blood transfusion. She was on the Triple drug “TLD” regimen (Tenofovir + Lamivudine + Dolutegravir) of ART for four years. Despite persistent complaints for two months, there was no history of previous Papanicolaou (PAP) smears taken. Her vitals were stable.

**Figure 1:**
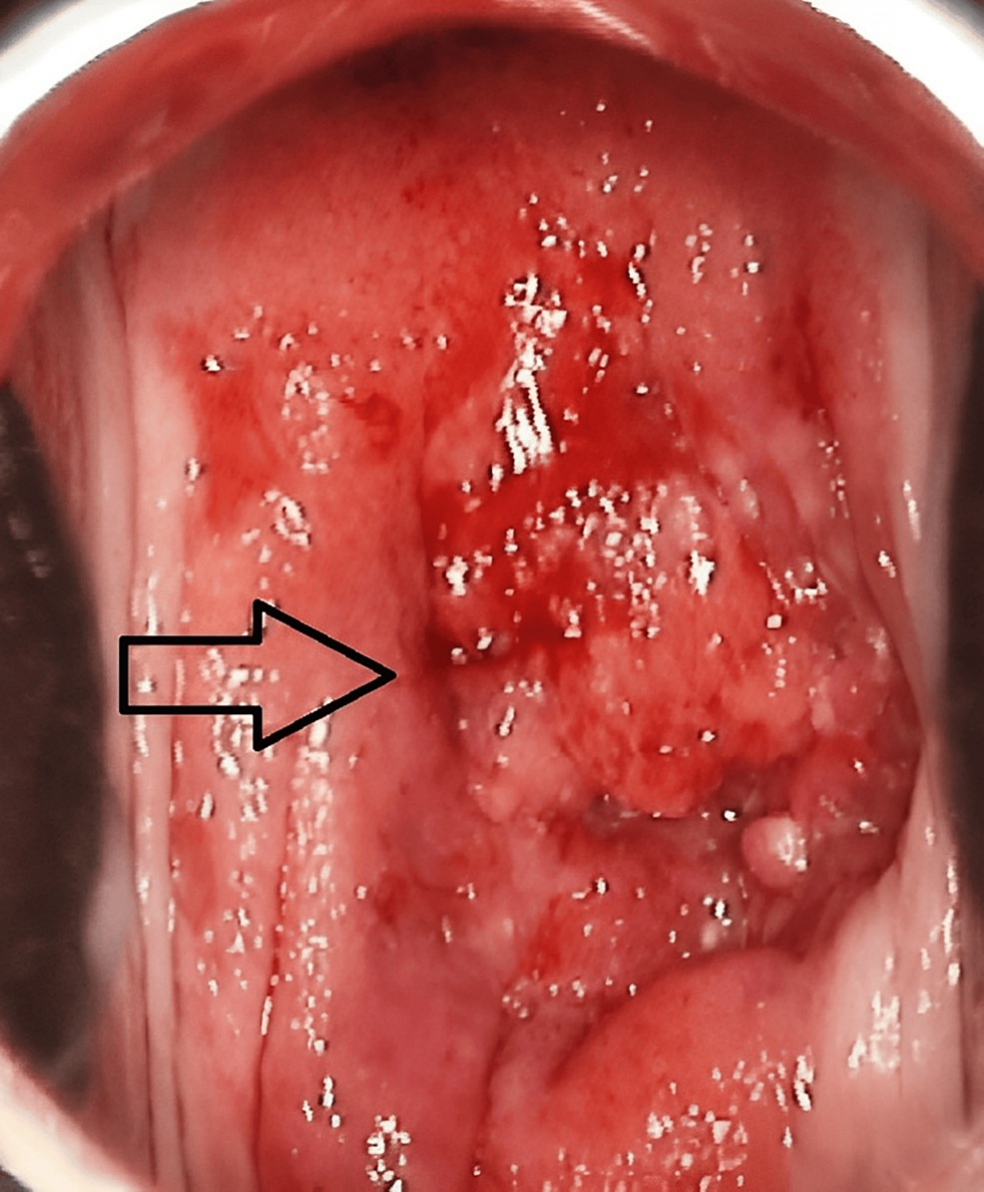
Ulcerative cervical lesions

Colposcopic per speculum examination revealed an endophytic cervical lesion which replaced the entire cervix without any apparent extra-cervical spread. Complete aceto-uptake was seen and an ulcerative lesion showing punctate vessels with bleeding (Figure 1) was observed.

**Figure 2:**
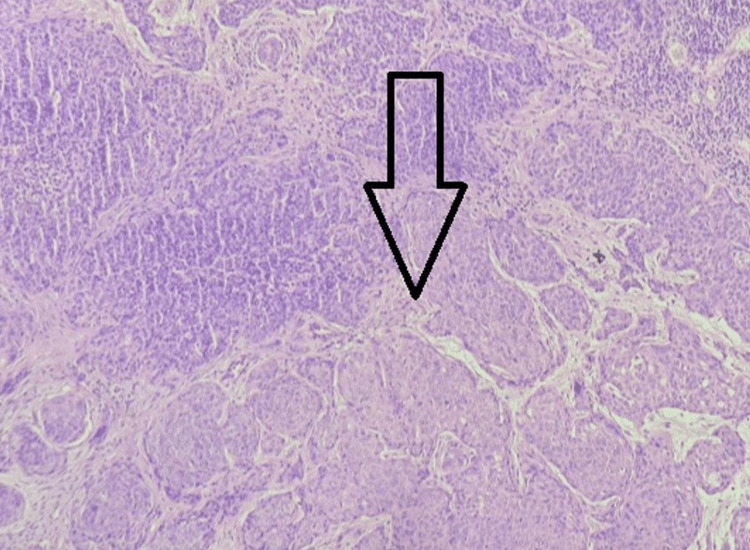
Histopathological features are suggestive of moderately differentiated squamous cell carcinoma of the cervix

On the application of Lugol’s iodine, the ectocervix visible was completely pale with few mosaics and white rings around gland openings, margins were blurred and there was the presence of coarse and rising terminal vasculature. On per vaginal examination, a hard growth was felt which obliterated the upper 2/3rd of the vagina. On per rectal examination, bilateral parametrium was apparently involved. On clinical examination, further supported by the results of the radiological scans done to check for distant metastases, a diagnosis of International Federation of Gynecology and Obstetrics (FIGO) Stage III-B cervical carcinoma was made as there was pelvic wall involvement and no distant spread, along with features suggestive of hydronephrosis or a non-functioning urinary tract. Under universal precautions, cervical biopsy was taken. On histopathological examination, the cervical biopsy was interpreted as moderately differentiated squamous cell carcinoma as shown in Figure 2. The section stained with hematoxylin and eosin shows round to irregularly shaped squamous cells, arranged in nests, medium-sized and nearly uniform cells with indistinct cell borders. The section also shows occasional scattered inflammatory infiltrates and congested blood vessels.

A cluster of differentiation (CD) 4 count was done, which turned out to be low (298/mm^3^). The CD4/CD8 ratio turned out to be 0.8 and the polymerase chain reaction Pause for quantitative HIV-RNA revealed a titer of 7368 copies/ml. A sample was sent for cartridge-based nucleic acid amplification test (CBNAAT) examination to rule out tuberculosis of the cervix, which turned out to be negative. As the patient presented in stage III b, a multidisciplinary approach with input from radiologists as well as oncologists was planned. Concurrent chemoradiation was decided as the best course of action. She was referred to the oncology center where she was managed with a combination of chemo and radiotherapy. Single-agent cisplatin was given as is standard protocol. Concurrent external beam radiotherapy and chemotherapy were given. Due to extensive involvement of the pelvis, brachytherapy was avoided. External beam radiotherapy (EBRT) using the box method or conformal method was given with a dose total dose of 48-50 Gy (gray) was decided upon as the course of action. Five cycles with a dose of 10 Gy per fraction were planned. Cisplatin was given once every week during the EBRT cycles. Periodic CD4 cell counts were done during the course of the treatment in order to ensure that the counts do not increase above 200 cells/mm^3^. A review to assess toxicity, with special emphasis on a complete blood count to assess anemia, neutropenia, and thrombocytopenia, was done every week, as the rate of hematological toxicity is known to be higher in those infected with HIV. All side effects like nausea, vomiting, diarrhea, and hair loss were explained to the patient. The patient was requested to follow up in the gynecology outpatient department after the completion of her chemoradiation cycles for regular examination and imaging as required.

## Discussion

Carcinoma of the cervix is the second most prevalent cancer affecting Indian women. Cervical intraepithelial neoplasia (CIN) occurs at a lower age, and if diagnosed at this stage, progression into carcinoma can be prevented [7]. In the early stages, invasive cervical carcinoma presents with no symptoms and can only be discovered either accidentally or via routine screening [8,9]. Cases associated with HIV have the prevalence of developing CIN four to five times more frequently [5]. Untreated CIN has the potential to develop into cervical cancer. The percentage of cervical cancer diagnoses among women who also have HIV is roughly 5% in high-income nations like the United States and certain other Western nations [9]. This is due to the existence of cutting-edge preventative recommendations, such as HIV testing for early detection and available treatments and dependable and efficient HPV vaccination screenings for cervical cancer. It is a unique feature of cervical carcinoma that most of its patients do not present early for treatment and even those who present early do not have the consolation of knowing that their growth is treatable as the duration of symptoms is not proportional to the extent of the disease. The common symptoms are leukorrhea, post-coital bleeding, and dyspareunia, as was the presentation in our case [10]. It is important to rule out tuberculosis of the cervix in low-resource settings as it can present with similar symptoms as invasive carcinoma [3,11]. Upon examination, the cardinal signs of the disease are hardness, friability, fixation, and bleeding on examination [4]. Any cervix that bleeds on touch is suspect. Cervical cytology is a good screening method to detect early symptomless invasive carcinoma. Colposcopic examination in the hands of experts can reveal carcinoma not apparent to the naked eye but its chief value is to indicate the most profitable sites from which biopsies can be taken in order to establish a diagnosis [12]. Cervical biopsy is the only method for definitive diagnosis and must be done in every case where carcinoma is suspected [13]. The most common pathological variant is squamous cell carcinoma of the cervix, which was also the diagnosis in our case. Unless a cone biopsy is taken, endocervical curettage is also necessary to exclude endocervical tumors. Treatment is dependent on the stage of carcinoma, as well as the general condition of the patient. As mentioned before, in the above case as the patient presented late and in an advanced stage, the only possible treatment was concurrent chemoradiation, which has a longer course, and increased economic burden [14].

Women who are infected with HIV have three times increased risk of cancer of the cervix than those not affected similarly [10]. Providing only antiretroviral treatment is not sufficient to improve the health of women infected by HIV. Any gain from HIV treatment regimens and programs is diminished if comorbidities such as the very high occurrence of cervical carcinoma and the subsequent morbidity and mortality caused by it among HIV-infected women are neglected [7]. According to current guidelines, women infected with HIV should receive PAP smears, first at the time of diagnosis, followed by another test at six months and annual smears in the subsequent period. But in spite of these recommendations, the screening rate for cervical carcinomas in HIV-infected women is inadequate which leads to diagnosis at advanced stages as seen in our case [2]. Prognosis and treatment are decided on the basis of FIGO staging, and once the tumor extends beyond the pelvic wall, all operative benefit is lost. Detection of carcinoma at an early stage can result in early operative intervention which can prevent significant morbidity and mortality amongst those affected by cervical carcinoma. This is especially true for immunocompromised individuals such as the one demonstrated in this case report. A multidisciplinary approach, as seen in our case is necessary especially in patients with HIV where factors such as CD4 counts, increased occurrence of toxicities, and interactions with antiretroviral drugs need to be considered. It is especially important to detect invasive carcinomas in the early stages in low-resource settings as the financial burden of the disease must be considered. The morbidity caused by advanced-stage carcinoma and its treatment is entirely avoidable if routine and vigorous screening is implemented especially in vulnerable cases like ours.

## Conclusions

As seen in our case, the diagnosis of carcinoma cervix may get delayed if symptoms like leukorrhea are overlooked both by the patient and the healthcare providers. Delay in diagnosis leads to a significant increase in morbidity and mortality, especially among those who are already vulnerable due to immunocompromised states such as HIV seropositive patients. The increased risk of toxicity due to chemoradiation and possible interactions of chemotherapeutic agents with antiretroviral drugs are added complications that make the management of such cases challenging. Hypervigilance regarding CD4 counts before, during, and after the course of treatment is vital, and the risk of mortality is ever present. Hence, women infected with HIV should be counseled regarding the symptoms of cervical carcinoma, so that they can report them to their healthcare providers at the earliest. The healthcare providers also need to be vigilant and take every opportunity to screen such patients in order to facilitate early diagnosis and treatment, so that cases of cervical carcinomas in these patients do not go overlooked.
